# A dose-ranging study of the inhaled dual phosphodiesterase 3 and 4 inhibitor ensifentrine in COPD

**DOI:** 10.1186/s12931-020-1307-4

**Published:** 2020-02-10

**Authors:** Dave Singh, Fernando J. Martinez, Henrik Watz, Thomas Bengtsson, Brian T. Maurer

**Affiliations:** 1 Medicines Evaluation Unit, University of Manchester & Manchester University NHS Foundation Trust, Manchester, UK; 2 Weill Cornell Medical College, New York, New York, and University of Michigan School of Medicine, Ann Arbor, MI USA; 3 Pulmonary Research Institute at Lung Clinic Grosshansdorf, Airway Research Center North (ARCN), Member of the German Center for Lung Research (DZL), Grosshansdorf, Germany; 4 StatMind AB, Lund, Sweden; 5 Verona Pharma plc, London, UK

**Keywords:** Phosphodiesterase inhibitors, Chronic obstructive pulmonary disease, Spirometry, Symptoms, Safety

## Abstract

**Background:**

Many patients with chronic obstructive pulmonary disease (COPD) still experience daily symptoms, exacerbations, and accelerated lung function decline, even when receiving maximal combined treatment with inhaled long-acting bronchodilators and corticosteroids. Novel treatment options are needed for these patients.

Phosphodiesterases (PDEs) are enzymes that impact a range of cellular functions by modulating levels of cyclic nucleotides, and there is evidence to suggest that combined inhibition of PDE3 and PDE4 can have additive (or perhaps synergistic) effects. This study investigated the efficacy and safety of ensifentrine, a first-in-class dual inhibitor of PDE 3 and 4, in patients with COPD.

**Methods:**

This randomised, double-blind, placebo-controlled, parallel-group, dose-ranging study recruited patients with COPD, post-bronchodilator forced expiratory volume in 1 s (FEV_1_) 40–80% predicted and FEV_1_/forced vital capacity ratio ≤ 0.7. Patients were randomised equally to inhale nebulised ensifentrine 0.75, 1.5, 3 or 6 mg or placebo, all twice daily. Primary outcome: placebo-adjusted difference in peak FEV_1_ (assessed over 3 h) at Week 4.

**Results:**

The study took place between July 2017 and February 2018. Of 405 patients randomly assigned to medication, 375 (92.6%) completed the study. For peak FEV_1_ at Week 4, all four ensifentrine doses were superior to placebo (*p* ≤ 0.0001) with least squares mean differences of 146 (95% CI 75–216), 153 (83–222), 200 (131–270) and 139 (69–210) mL for ensifentrine 0.75, 1.5, 3 and 6 mg, respectively. Respiratory symptoms (assessed using the Evaluating Respiratory Symptoms questionnaire) were also significantly improved with all ensifentrine doses at Week 4. Adverse events were reported by 33.3, 44.4, 35.4 and 36.3% patients with ensifentrine 0.75, 1.5, 3 and 6 mg, respectively, and 39.2% with placebo.

**Conclusions:**

In this four-week Phase IIb study, all four ensifentrine doses significantly improved bronchodilation and symptoms, with a dose-ranging effect from 0.75 to 3 mg twice daily, and all doses well tolerated. The study supports the continuing development of ensifentrine in COPD.

**Trial registration:**

EudraCT 2016–005205-40, registered 30 May 2017.

## Background

Characteristic features of chronic obstructive pulmonary disease (COPD) are airflow obstruction and persistent inflammation [1]. Inhaled long-acting bronchodilators and corticosteroids have been shown to improve symptoms and health-related quality of life, and to reduce exacerbation rates in patients with COPD [1]. However, many patients still experience daily symptoms [2–4], exacerbations [5–7], and accelerated lung function decline [8], even when receiving maximal combined treatment with these inhaled drugs. Novel treatment options are needed for these patients.

Phosphodiesterases (PDEs) are enzymes that impact a range of cellular functions by modulating levels of cyclic nucleotides. PDE3 regulates cyclic adenosine monophosphate (cAMP) and cyclic guanosine monophosphate (cGMP) concentrations in airway smooth muscle, such that inhibition results in airway smooth muscle relaxation [9–11]. PDE4 regulates cAMP concentrations and is involved in inflammatory cell activation; consequently, inhibition has anti-inflammatory effects [9–11]. There is evidence to suggest that combined inhibition of PDE3 and PDE4 can have additive (or perhaps synergistic) effects with respect to both anti-inflammatory and bronchodilator activity [9].

Ensifentrine is an inhaled first-in-class dual inhibitor of PDE3 and PDE4 that has previously demonstrated bronchodilator and anti-inflammatory efficacy in proof of concept studies in healthy volunteers, and in patients with asthma or COPD [12–15]. In patients with COPD, lung function and safety data had previously been obtained from up to 6 days treatment, although with no evaluation of symptoms [15]. Delivery of ensifentrine by inhalation aims to minimise the systemic adverse effects that have been associated with oral administration of PDE inhibitors [16].

This randomised, double-blind, placebo-controlled, parallel-group Phase IIb study aimed to investigate the efficacy, in terms of lung function and symptoms, and safety of 4 weeks dosing of a range of ensifentrine doses in patients with COPD who did not receive any concomitant long-acting bronchodilator therapy for COPD.

## Methods

### Study design

Patients meeting the inclusion/exclusion criteria at screening entered a 7–14-day run-in period during which long acting bronchodilator therapy was washed out; inhaled corticosteroids were continued at the same dose from ≥4 weeks prior to screening and throughout the study (Additional file 1: Figure S1). After the run-in, patients were randomised equally to five treatment groups, to inhale twice daily nebulised ensifentrine 0.75, 1.5, 3 or 6 mg or placebo. Patients then attended the study centre weekly for 4 weeks. Rescue salbutamol was permitted, but not within 8 h before spirometry assessments.

The study was approved by independent ethics committees at each institution, and was performed in accordance with the Declaration of Helsinki and Good Clinical Practice (ICH/CPMP/135/95). The study is registered at ClinicalTrials.gov (NCT03443414) and EudraCT (2016–005205-40).

### Patients

Eligible patients were: males or females aged 40–75 years, inclusive; diagnosed with COPD for at least 1 year; clinically stable for at least 4 weeks; post-bronchodilator forced expiratory volume in 1 s (FEV_1_) 40–80% predicted normal and FEV_1_/forced vital capacity ratio ≤ 0.7; and current or former smokers with smoking history of at least 10 pack-years. To remain eligible, pre-dose FEV_1_ at randomisation was to be ±20% of the screening value. All patients provided written informed consent prior to any study-related procedure. Full inclusion and exclusion criteria are in the additional file.

### Procedures

At the randomisation visit, baseline (pre-dose) data were collected for spirometry (FEV_1_), St. George’s Respiratory Questionnaire – COPD Specific (SGRQ-C), Baseline Dyspnoea Index, and Medical Research Council dyspnoea scale (MRC). Spirometry was assessed pre-dose and up to 3 h post-dose on Weeks 1, 2 and 3, and up to 12 h post-dose on Day 1 and Week 4. At Week 4, Transition Dyspnoea Index (TDI), SGRQ-C, MRC and patient’s global assessment of change were assessed pre-dose. The patient’s global assessment of change is a study-specific questionnaire in which patients were asked, “Compared with prior to the study start, how do you feel your breathing is?”, graded on a scale of 1 = much worse to 5 = much better, with 3 = no change. Daily throughout the study (including between screening and randomisation) patients used an e-diary to record rescue medication use and COPD symptoms (using the Evaluating Respiratory Symptoms [E-RS™:COPD] questionnaire). Vital signs (blood pressure and pulse rate) and 12-lead electrocardiograms were assessed pre- and post-dose on all visits, with Holter monitor data collected over the 24-h periods prior to randomisation (for baseline) and the Week 4 visit. Adverse events were captured over the study duration.

### Outcomes

The primary objective was to investigate the placebo-corrected effect of ensifentrine on change from baseline in peak FEV_1_ (assessed over 0–3 h) on Week 4. Secondary efficacy variables were: morning trough FEV_1_ (measured 15 min pre-dose) on Weeks 1–4; average FEV_1_ over 0–12 h on Day 1 and Week 4; average FEV_1_ over 0–3 h on Day 1 and Weeks 1–4; peak FEV_1_ on Day 1 and Weeks 1–3; E-RS™:COPD total score and average daily use of rescue medication over Weeks 1, 2, 3 and 4; and TDI, SGRQ-C total score (mean and percentage of responders), MRC and patient’s global assessment of change after 4 weeks. Ensifentrine safety was assessed by adverse events, electrocardiogram data, Holter monitoring, laboratory safety tests and vital signs.

### Randomisation and masking

Patients were assigned to treatment groups in accordance with a randomisation list generated by the sponsor’s contract research organisation. Patients, investigators, site staff and sponsor personnel were masked to treatment assignment for the duration of the study, with the exceptions of the sponsor’s clinical supply chain lead, and of designated unblinded personnel at the sites, who, because the active and placebo treatments did not visually match, administered the in-clinic dose to patients and were not to take part in any of the study assessments.

### Statistical analysis

Peak FEV_1_ standard deviation was estimated to be 250 mL. With a two-sided test at a 5% significance level and 80 evaluable patients per group, it was estimated that there would be 80% power to detect a true difference of 111 mL between any two treatments. This detectable limit was considered sufficient to identify a minimal effective dose of ensifentrine. Thus 80 patients per group were to be randomised.

The primary endpoint (Week 4 peak FEV_1_) was analysed using a restricted maximum likelihood-based mixed model for repeated measured (MMRM), including fixed effects for treatment, visit and treatment by visit interaction, patient as random effect, baseline value as covariate and covariance structure by visit. Ensifentrine–placebo differences with 95% confidence intervals and corresponding two-sided *p*-values were calculated. To control for the familywise error rate, a fixed-sequence testing strategy was employed, with the highest ensifentrine dose tested vs placebo. If a statistically significant difference was found at the two-sided α level of 5%, the testing proceeded with the next lower dose. If a test was non-significant, testing stopped and the remaining null hypotheses accepted.

A similar mixed model for repeated measured (MMRM) method was used to analyse most of the secondary efficacy endpoints, with the same hierarchical testing within endpoint, although endpoints were tested independently. The average FEV_1_ endpoints were calculated using the linear trapezoidal method as the area under the curve divided by the length of the time interval of interest. E-RS™:COPD total score data and rescue medication use were averaged to give weekly scores. The percentage of SGRQ-C responders, defined as patients with an improvement from baseline of ≥4 units, was analysed using a logistic regression model adjusting for treatment and country and giving the odds for being a responder as outcome, treatment differences expressed as the odds ratio.

The efficacy data were analysed in the full analysis set, which comprised all randomised patients who received at least one dose of study medication and had at least one post-treatment efficacy assessment. Safety analyses were in the safety population, which was all randomised patients who received at least one dose of study medication.

## Results

**Fig. 1:**
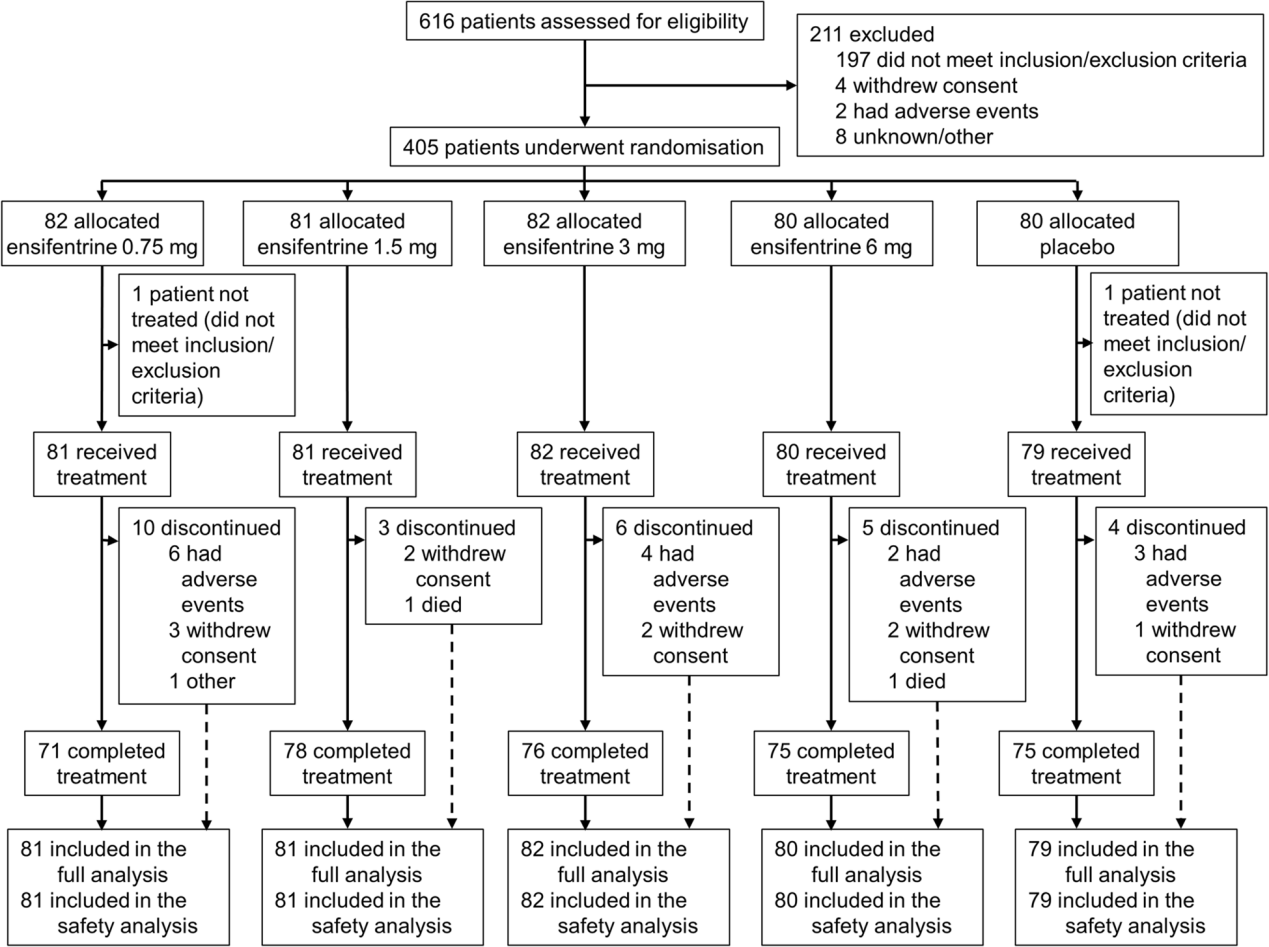
Patient flow through the study

The study took place between 10 July 2017 and 7 February 2018 in 47 study centres across six countries (Bulgaria [8], Czech Republic [2], Germany [16], Poland [13], Romania [7], and United Kingdom [1]). We recruited 616 patients, of whom 405 were eligible to be randomly assigned to medication, with 375 (93%) completing the study (Fig. 1). Compliance to treatment was high, with the median duration of exposure being 29.0 days in all five groups. Baseline characteristics of the recruited patients are shown in Table 1.

**Table 1:** Baseline demographics and disease characteristics (all randomised analysis set)

	Ensifentrine				Placebo (*N* = 80)
	0.75 mg (*N* = 82)	1.5 mg (*N* = 81)	3 mg (*N* = 82)	6 mg (*N* = 80)	
Age, years	63.6 (7.05)	63.4 (6.40)	62.5 (6.51)	62.9 (6.73)	63.5 (6.44)
Sex, n (%)					
Male	56 (68)	46 (57)	45 (55)	48 (60)	50 (63)
Female	26 (32)	35 (43)	37 (45)	32 (40)	30 (38)
Race, n (%)					
White	82 (100)	81 (100)	82 (100)	80 (100)	80 (100)
Post-bronchodilator FEV_1_					
L	1.67 (0.464)	1.60 (0.466)	1.62 (0.441)	1.63 (0.474)	1.69 (0.493)
% predicted	56.0 (10.34)	56.0 (9.83)	55.6 (10.18)	55.3 (9.47)	56.0 (9.89)
FEV_1_ reversibility, %	10.4 (10.13)	11.0 (12.32)	12.9 (13.53)	12.3 (11.14)	11.7 (10.60)
Smoking status, n (%)					
Current	50 (61)	40 (49)	47 (57)	42 (53)	43 (54)
Ex	32 (39)	41 (51)	35 (43)	38 (48)	37 (46)
Smoking history, pack-years	44.7 (21.27)	43.7 (21.98)	41.8 (19.05)	37.3 (16.75)	43.3 (20.21)
Chronic bronchitis, n (%)	48 (59)	43 (53)	56 (68)	58 (73)	46 (58)
SGRQ-C	49.9 (17.36)	43.4 (17.06)	42.1 (18.78)	44.1 (15.02)	42.3 (17.07)
BDI	5.9 (1.44)	6.4 (1.81)	6.4 (1.43)	6.4 (1.16)	6.4 (1.38)
MRC	2.8 (0.82)	2.5 (0.79)	2.5 (0.79)	2.6 (0.73)	2.5 (0.75)
MRC score, n (%)					
< 2	4 (5)	8 (10)	6 (7)	4 (5)	4 (5)
≥ 2	78 (95)	73 (90)	76 (93)	76 (95)	76 (95)
Rescue medication, puffs per day^a^	1.5 (1.80)	1.5 (1.84)	1.9 (2.14)	1.9 (2.13)	1.5 (1.88)
E-RS:COPD™^b^	13.6 (6.77)	12.3 (6.05)	12.0 (6.03)	12.2 (6.29)	11.5 (6.23)
Concomitant ICS use, n (%)	33 (40)	36 (44)	29 (35)	32 (40)	28 (35)

Data are mean (standard deviation) unless specified otherwise

^a^Available baseline data from 81, 81, 82, 80 and 79 patients, respectively

^b^Available baseline data from 75, 75, 77, 77 and 77, respectively

*Abbreviations*: *FEV*_*1*_ forced expiratory volume in 1 s, *SGRQ-C* St George’s Respiratory Questionnaire – COPD Specific, *BDI* Baseline Dyspnoea Index, *MRC* Medical Research Council dyspnoea scale, *E-RS:COPD™* Evaluating Respiratory Symptoms in COPD questionnaire, *COPD* chronic obstructive pulmonary disease, *ICS* inhaled corticosteroid

**Fig. 2:**
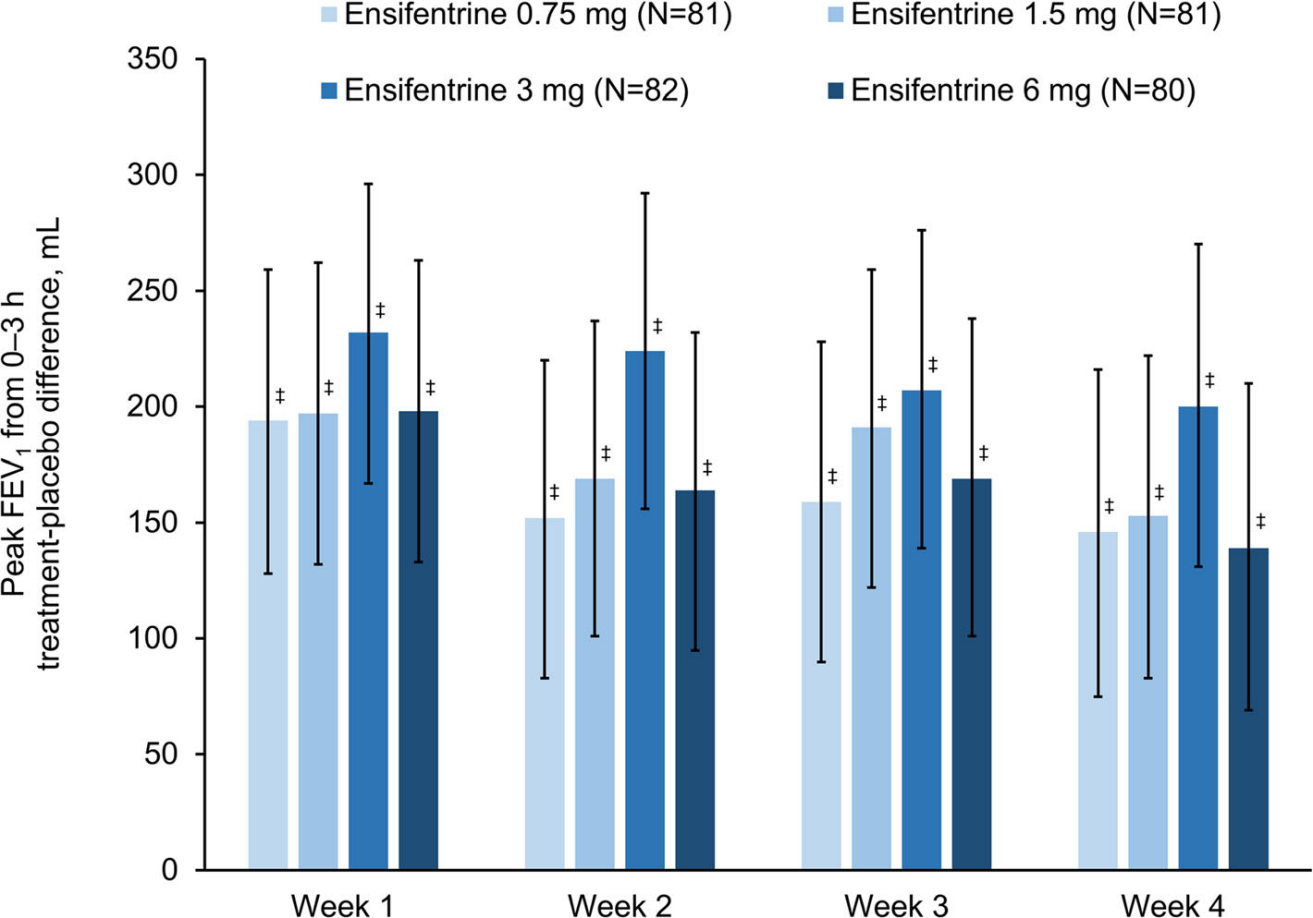
Peak FEV_1_ between 0 and 3 h post-dose (full analysis set).* Data are least squares means treatment–placebo differences and 95% confidence intervals.* ^*‡*^*p < 0.001* vs *placebo. Least squares mean changes from baseline in the placebo group (N = 79) were 36, 50, 42 and 57 mL at Weeks 1, 2, 3 and 4, respectively. Abbreviation: FEV*_*1*_*, forced expiratory volume in 1 s*

**Fig. 3:**
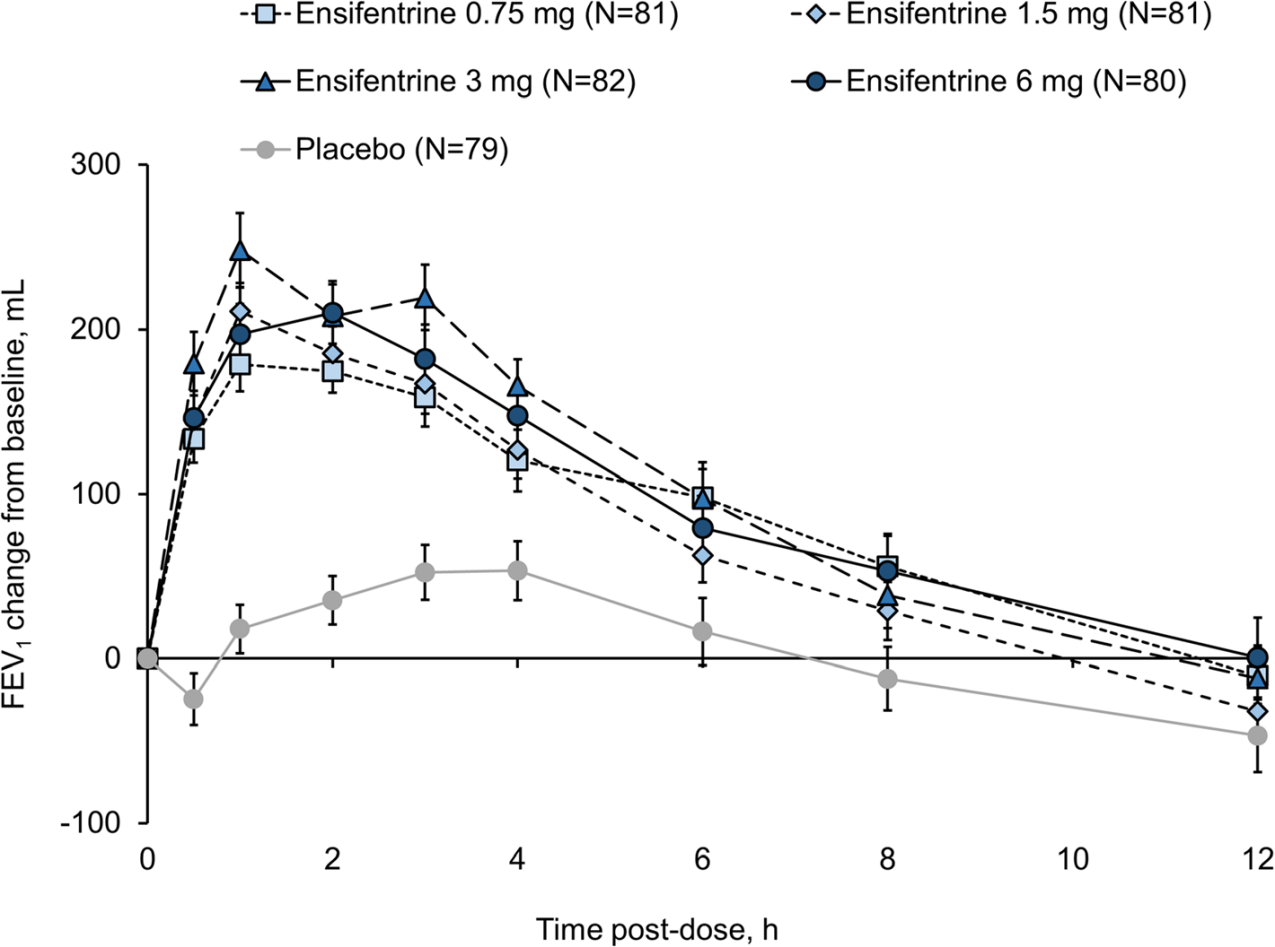
Individual timepoint FEV_1_ on Day 1 (full analysis set). *Data are mean and standard error of the mean. Abbreviation: FEV*_1_, *forced expiratory volume in 1 s*

**Fig. 4:**
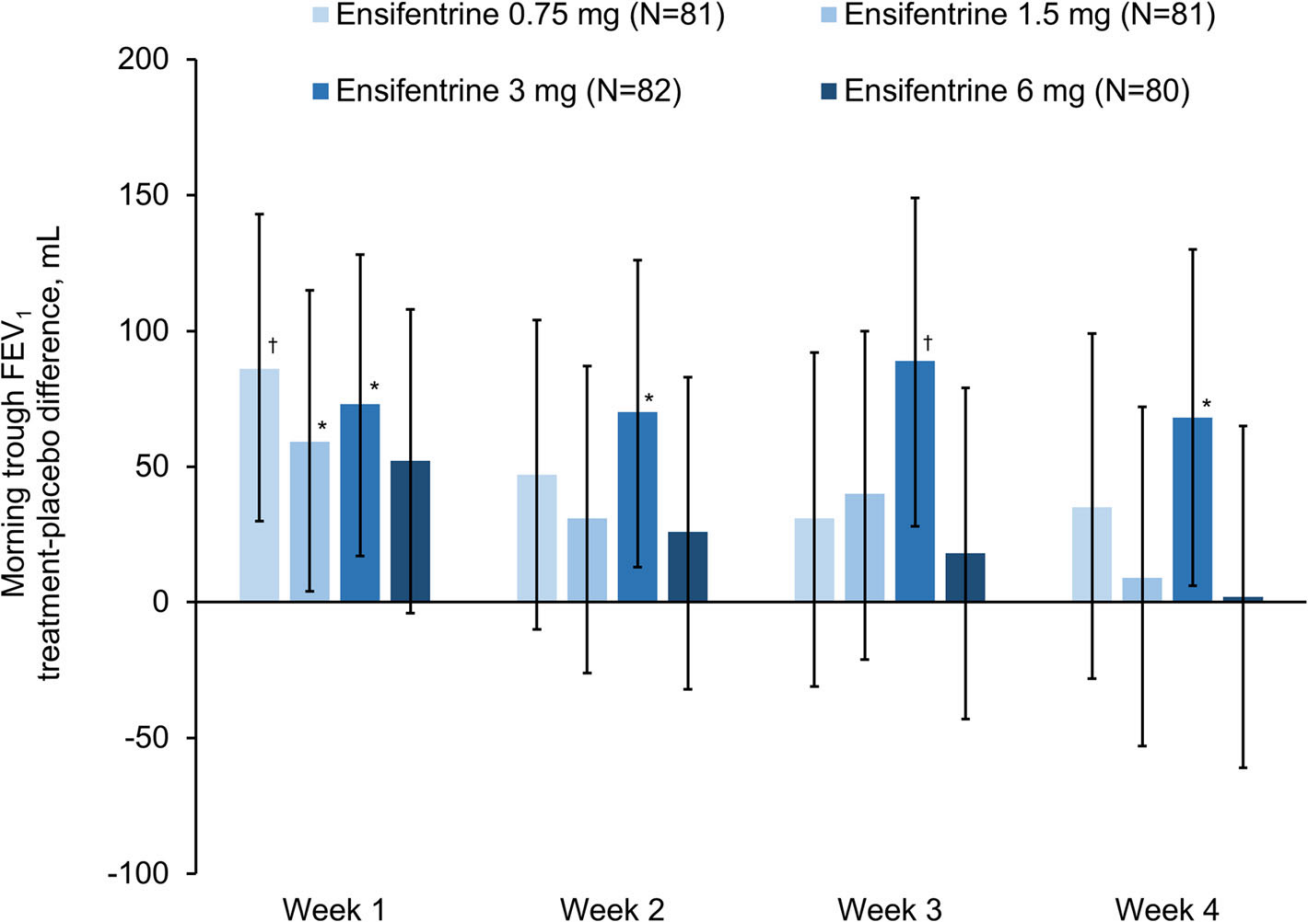
Morning trough FEV_1_ (full analysis set). Data are least squares means treatment–placebo differences and 95% confidence intervals. *p < 0.05; ^†^*p < 0.01* vs *placebo. Least squares mean changes from baseline in the placebo group (N = 79) were −56, −32, −49 and −28 mL at Weeks 1, 2, 3 and 4, respectively*

**Fig. 5:**
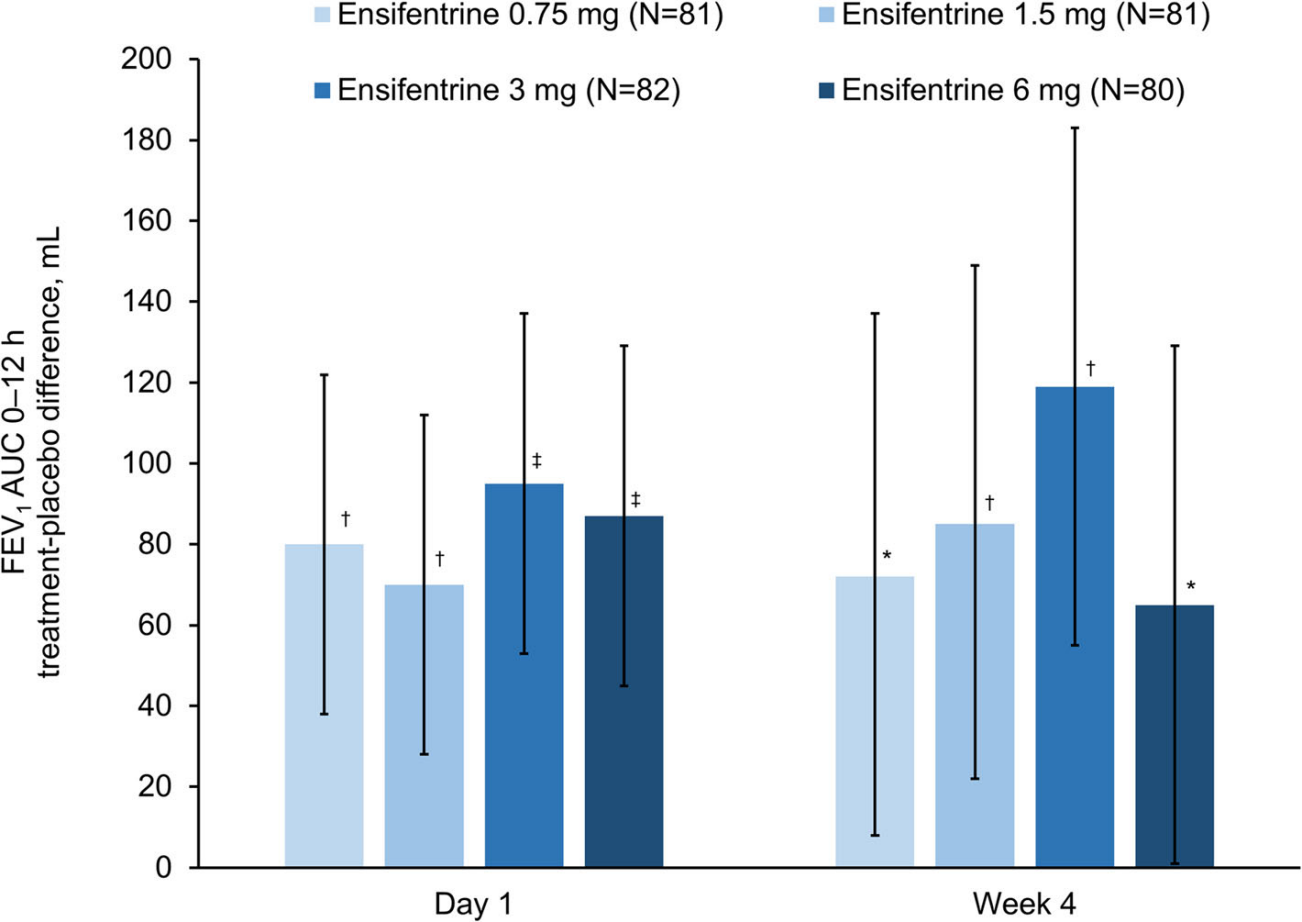
Average FEV_1_ over 0–12 h (full analysis set). Data are least squares means treatment–placebo differences and 95% confidence intervals. *p < 0.05; ^†^*p < 0.01;* ^‡^*p < 0.001 vs placebo. Least squares mean changes from baseline in the placebo group (N = 79) were 8 and −33 mL on Day 1 and at Week 4, respectively. Abbreviation: FEV*_1_*, forced expiratory volume in 1 s*

For the primary endpoint (peak FEV_1_ on Week 4), all four ensifentrine doses were superior to placebo (*p* < 0.001) with differences between 139 and 200 mL (Fig. 2). Following the first dose on Day 1, there was an immediate increase in FEV_1_ in all four ensifentrine groups (Fig. 3), with peak FEV_1_ on Day 1 significantly higher for all ensifentrine doses compared to placebo, and differences vs placebo of 153 (95% confidence interval 105, 201), 158 (110, 206), 207 (159, 256), and 173 (125, 221) mL for ensifentrine 0.75, 1.5, 3 and 6 mg (all *p* < 0.001). Peak FEV_1_ was also significantly higher for all ensifentrine doses compared to placebo after 1, 2 and 3 weeks (p < 0.001; Fig. 2), whereas for morning (pre-dose) trough FEV_1_, only ensifentrine 3 mg was consistently statistically superior to placebo, with differences ranging between 68 and 89 mL (Fig. 4). Average FEV_1_ over 12 h for all ensifentrine doses was superior to placebo, both immediately following the first dose (Day 1) and after four weeks, for example with a difference vs placebo of 119 mL for the 3 mg dose at Week 4 (Fig. 5). Similarly, average FEV_1_ over 0–3 h for all ensifentrine doses was superior to placebo at all visits (Additional file 1: Table S1). In general, a dose-response effect on FEV_1_ parameters was apparent for ensifentrine doses between 0.75 mg and 3 mg.

**Fig. 6:**
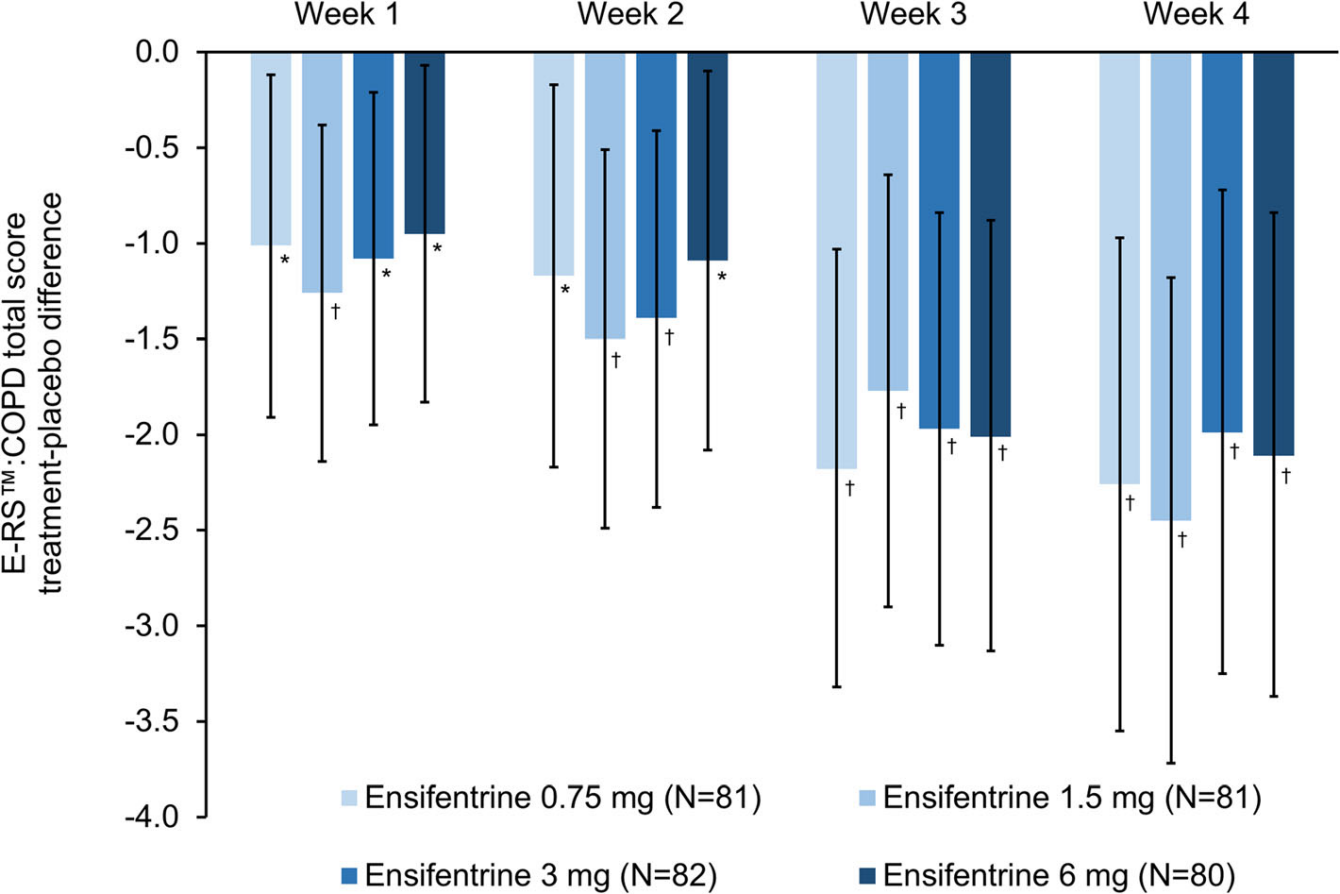
E-RS™:COPD total score (full analysis set). Data are least squares means treatment–placebo differences and 95% confidence intervals. *p < 0.05; ^†^*p < 0.01 vs placebo. Least squares mean changes from baseline in the placebo group (N = 79) were 0.38, 0.57, 1.11 and 1.19 at Weeks 1, 2, 3 and 4, respectively. Abbreviation: ERS:COPD™, Evaluating Respiratory Symptoms in COPD questionnaire*

There was a progressive improvement in symptoms over the duration of the study for all four doses compared to placebo as measured by daily E-RS™:COPD total score (Fig. 6). For TDI focal score, all four ensifentrine doses were superior to placebo at Week 4, with differences ranging from 1.11 to 1.64 (Table 2). Compared with patients receiving placebo, after four weeks there was a numerical improvement vs placebo in both mean SGRQ-C total score and the percentage of responders with all four ensifentrine doses, although the differences vs placebo were not statistically significant (Table 2 and Additional file 1: Table S2). Similarly, at Week 4 there was a numerical improvement vs placebo in MRC total score for all ensifentrine doses (Additional file 1: Table S3), whereas for the patient’s global assessment of change, all ensifentrine doses were statistically superior to placebo at this timepoint (Additional file 1: Table S3). Rescue medication use was numerically lower with all four ensifentrine doses compared with placebo over all four periods, with the reduction statistically significant for the 1.5, 3 and 6 mg doses in the Week 2, 3 and 4 analyses (Additional file 1: Table S4).

**Table 2:** TDI focal score and SGRQ-C total score after 4 weeks (full analysis set)

Ensifentrine dose	TDI focal score		SGRQ-C total score	
	Mean (SD)	Treatment–placebo difference (95% confidence interval); *p* value	Mean (SD)	Treatment–placebo difference (95% confidence interval); *p* value
6 mg (*N* = 80)	1.39 (2.179)	1.11 (0.16 to 2.06); 0.022	41.5 (15.20)	−2.67 (− 6.26 to 0.91); 0.143
3 mg (*N* = 82)	1.55 (3.436)	1.19 (0.25 to 2.14); 0.014	40.1 (15.93)	−2.29 (−5.96 to 1.37); 0.220
1.5 mg (*N* = 81)	1.92 (3.221)	1.64 (0.69 to 2.59); 0.001	41.4 (16.24)	−2.85 (−6.46 to 0.76); 0.121
0.75 mg (*N* = 81)	1.49 (2.810)	1.29 (0.32 to 2.25); 0.009	45.1 (14.95)	−2.22 (−5.87 to 1.42); 0.231
Placebo (*N* = 79)	0.37 (3.220)		43.5 (16.99)	

Abbreviations: TDI, Transition Dyspnoea Index; SGRQ-C, St George’s Respiratory Questionnaire – Chronic Obstructive Pulmonary Disease Specific

The overall proportion of patients experiencing adverse events was similar with all five treatments (Table 3). Occurrence was not related to ensifentrine dose, and the majority of events were mild or moderate in severity. The only adverse events considered related to study medication that occurred in more than two patients with any treatment were cough, dyspnoea and productive cough; again, occurrence was not related to ensifentrine dose (Table 3). Seven patients had a serious adverse event (Table 3), only two of whom experienced events considered by the investigators to be related to study medication: one patient receiving ensifentrine 1.5 mg, who committed suicide (although the patient had significant personal and financial stress factors); and one patient receiving ensifentrine 0.75 mg who had a medical history of hepatic cirrhosis (and so did not meet study eligibility criteria), and who experienced haemorrhage of oesophageal varices (which resolved after study medication was interrupted), hepatic cirrhosis and hepatic encephalopathy. A second patient died during the study, with the death being of unknown cause, but considered unrelated to study medication.

**Table 3:** Adverse events, overall and most common (safety analysis set)

	Ensifentrine				Placebo (*N* = 79)
	0.75 mg (*N* = 81)	1.5 mg (*N* = 81)	3 mg (*N* = 82)	6 mg (*N* = 80)	
Any adverse event	27 (33)	36 (44)	29 (35)	29 (36)	31 (39)
Headache	4 (5)	4 (5)	7 (9)	4 (5)	3 (4)
Worsening of COPD symptoms	5 (6)	5 (6)	3 (4)	3 (4)	6 (8)
Cough	4 (5)	4 (5)	6 (7)	1 (1)	1 (1)
Nasopharyngitis	2 (2)	4 (5)	4 (5)	5 (6)	7 (9)
Hypertension	2 (2)	1 (1)	4 (5)	3 (4)	1 (1)
Nausea	3 (4)	2 (2)	2 (2)	0	2 (3)
Dyspnoea	3 (4)	1 (1)	1 (1)	1 (1)	5 (6)
Productive cough	0	3 (4)	1 (1)	0	0
Any treatment-related adverse event	8 (10)	11 (14)	12 (15)	8 (10)	10 (13)
Cough	2 (2)	1 (1)	4 (5)	1 (1)	1 (1)
Dyspnoea	1 (1)	0	0	0	3 (4)
Productive cough	0	3 (4)	0	0	0
Any severe adverse event	4 (5)	1 (1)	2 (2)	1 (1)	2 (3)
Any serious adverse event	2 (2)	2 (2)	1 (1)	1 (1)	1 (1)
Any serious treatment-related adverse event	1 (1)	1 (1)	0	0	0
Any adverse event leading to drug discontinuation	6 (7)	1 (1)	4 (5)	2 (3)	2 (3)
Any adverse event leading to death	0	1 (1)	0	1 (1)	0

Data are n (%). The most common adverse events and drug-related adverse events are those reported in more than two patients in any group

There was no relationship between ensifentrine dose and adverse events leading to withdrawal from the study. Six patients withdrew from the study due to adverse events considered related to study medication: three patients in the ensifentrine 0.75 mg group (hepatic cirrhosis [one patient]; vomiting, headache and nausea [one patient]; dyspnoea [one patient]); one patient in the ensifentrine 1.5 mg group (suicide); and one patient each in the ensifentrine 6 mg and placebo groups (worsening of COPD symptoms).

Changes from baseline in electrocardiogram parameters were minimal, and were similar across treatment groups, with no electrocardiogram-related serious adverse events or adverse events leading to premature withdrawal. Overall, no trends by treatment or clinically meaningful changes were observed in any Holter electrocardiogram parameter during the study. Finally, there were no treatment-related trends in any of the laboratory tests or vital sign parameters.

## Discussion

Overall, all four ensifentrine doses were more effective than placebo, with a dose-dependent response between 0.75 and 3 mg for peak and average FEV_1_. Bronchodilation was apparent from the first dose, with all ensifentrine doses providing immediate improvements in lung function from Day 1, and minimal further improvements in ensifentrine–placebo differences from Week 1 to Week 4. Despite being only a four-week study, ensifentrine also provided symptomatic improvements versus placebo in terms of E-RS™:COPD total score and TDI focal score. Differences versus placebo for all four ensifentrine doses at Week 4 were close to, or exceeded, the E-RS™:COPD total score minimum clinically important difference (MCID) of 2 units, and exceeded the TDI focal score MCID of 1 unit. Whereas the effects of ensifentrine on bronchodilation were apparent from the first dose, there was a gradual improvement in symptoms (E-RS™:COPD). This further highlights the often poor association between FEV_1_ and patient reported outcomes, and in this case indicates that the improvement of persistent symptoms follows a gradual time course rather than a rapid improvement. Longer studies are planned to further evaluate this progressive symptomatic improvement.

The rapid bronchodilation observed is consistent with previous short-term ensifentrine studies in COPD, and with preclinical evidence of the direct effect of ensifentrine on large and small airways, including increased mucociliary clearance and bronchial relaxation [17–19]. In particular, ensifentrine had a substantial effect on peak FEV_1_, with differences vs placebo at Week 4 between 139 and 200 mL; these improvements were consistent with the effects on FEV_1_ averaged over 12 h. The more progressive improvement in symptoms (especially breathlessness) could, at least in part, be due to a reduction in hyperinflation. Indeed, in a number of studies of inhaled long-acting bronchodilators, maximal improvements in FEV_1_ parameters were observed early in the study, whereas improvements in hyperinflation tended to be more progressive, with the maximal effect not observed for several weeks [20, 21]. In previous short-term studies, ensifentrine significantly reduced residual volume, a marker of hyperinflation, compared with placebo, and the addition of ensifentrine to salbutamol, ipratropium or tiotropium significantly reduced residual volume compared with salbutamol, ipratropium or tiotropium alone [13]. Although not assessed in the current study, improvements in residual volume are not detected by FEV_1_ changes, and are likely to have occurred in at least a subgroup of patients. Another factor contributing to the symptomatic improvement, and also explaining the gradually increasing effect on these endpoints, could be the anti-inflammatory effect of ensifentrine, as demonstrated in preclinical studies [18] and in a clinical study [12], although again this was not directly assessed here.

The lung function data suggest a dose-response up to 3 mg that plateaus at higher doses (6 mg, although this dose was still effective). This lung function dose-response was not mirrored in the symptom data, where all ensifentrine doses showed similar effects on symptoms measured by TDI and E-RS™:COPD, and with the ensifentrine 6 mg dose not worse than 3 mg for these endpoints. A previous crossover study of 3 days treatment with ensifentrine in addition to tiotropium showed that 6 mg had greater effects compared to 1.5 mg on lung function [13]. However, in a six-day parallel-group study the lung function effects of ensifentrine 6 mg did not differentiate from 1.5 mg [15]. These differing lung function results may reflect more between-patient variability in lung function responses that can occur in parallel-group studies. Overall, the available data suggest that ensifentrine doses of 3 and 6 mg are likely to be at the top of the dose-response curves.

This study was not powered to show statistically significant improvements in the MRC dyspnoea scale or in the SGRQ-C total score or responder analysis, even though there were clear numerical improvements in these parameters versus placebo. The MRC is relatively insensitive to change [22], and it is possible that the study duration was insufficient to demonstrate a clear impact on SGRQ-C. Importantly, however, all four ensifentrine doses demonstrated a good overall safety and tolerability profile, with no dose-limiting safety signals observed. The overall frequency of adverse events was no greater with any ensifentrine dose than placebo, and the incidence of treatment-related adverse events was low. Importantly for this novel class of therapy, no gastrointestinal or cardiovascular treatment-related adverse events were observed. Furthermore, there were no treatment-related trends for any of the laboratory tests or vital sign parameters.

A limitation of the current study is the relatively short treatment duration. Although the differential effect of ensifentrine on lung function and symptoms in this study may suggest the presence of anti-inflammatory activity (with an immediate improvement in lung function, but a delayed impact on symptoms), the four-week study duration was insufficient to fully evaluate the anti-inflammatory effect of ensifentrine, especially on exacerbations – or to distinguish between bronchodilator and anti-inflammatory effects. Furthermore, although we did not apply any inclusion criteria to select patients with symptomatic COPD (other than requiring patients to be clinically stable), we acknowledge that the majority of the patients recruited had relatively high symptom scores (approximately 95% had MRC ≥2 at baseline), and so the use of one or two long-acting bronchodilators would be indicated. The current study did not evaluate the effect of ensifentrine in combination with such therapies, or compare ensifentrine to other treatments. However, the use of ensifentrine in combination with other bronchodilators has been evaluated in previous short-term studies in patients with COPD [13]. In these earlier studies, the addition of a single dose of ensifentrine 6 mg to salbutamol increased peak FEV_1_ by 108 mL compared with salbutamol alone (*p* < 0.01), and the addition of ensifentrine to ipratropium increased peak FEV_1_ by 94 mL (*p* < 0.01). Furthermore, after 3 days of treatment, the addition of ensifentrine 1.5 mg or 6 mg to tiotropium increased peak FEV_1_ by 104 and 127 mL, respectively (both *p* < 0.01).

In conclusion, in this four-week Phase IIb study all four doses of ensifentrine significantly improved bronchodilation and symptoms compared with placebo, with a dose-response from 0.75 to 3 mg twice daily, and all doses being well tolerated. Overall, the results suggest that ensifentrine has a novel dual mechanism of action and support the continuing development of ensifentrine in COPD.

## Supplementary information

**Additional file 1: Figure S1**. Study design. **Table S1**. Average FEV_1_ over 0–3 h (full analysis set). **Table S2**. Percentage of patients with ≥4 unit improvement in SGRQ-C total score after four weeks (full analysis set). **Table S3**. Medical Research Council dyspnoea score and patient global assessment of change after four weeks (full analysis set). **Table S4**. Rescue medication use (full analysis set).

## Data Availability

The data from this study are available on request, following submission of a valid research protocol to the corresponding author.
