# Supplementary material for: A dose-ranging study of the inhaled dual phosphodiesterase 3 and 4 inhibitor ensifentrine in COPD
Source: Respir Res. 2020 Feb 10;21:47. doi: 10.1186/s12931-020-1307-4 (PMC7011474; doi:10.1186/s12931-020-1307-4)
Supplement: Supplementary file 1 — Additional file 1: Figure S1. Study design. Table S1. Average FEV1 over 0–3 h (full analysis set). Table S2. Percentage of patients with ≥4 unit improvement in SGRQ-C total score after four weeks (full analysis set). Table S3. Medical Research Council dyspnoea score and patient global assessment of change after four weeks (full analysis set). Table S4. Rescue medication use (full analysis set). [file 12931_2020_1307_MOESM1_ESM.docx]

# A dose-ranging study of the inhaled dual phosphodiesterase 3 and 4 inhibitor ensifentrine in COPD

Dave Singh, Fernando J. Martinez, Henrik Watz, Thomas Bengtsson, Brian T. Maurer

# Additional file

# Methods

## Study design

Figure S1. Study design.


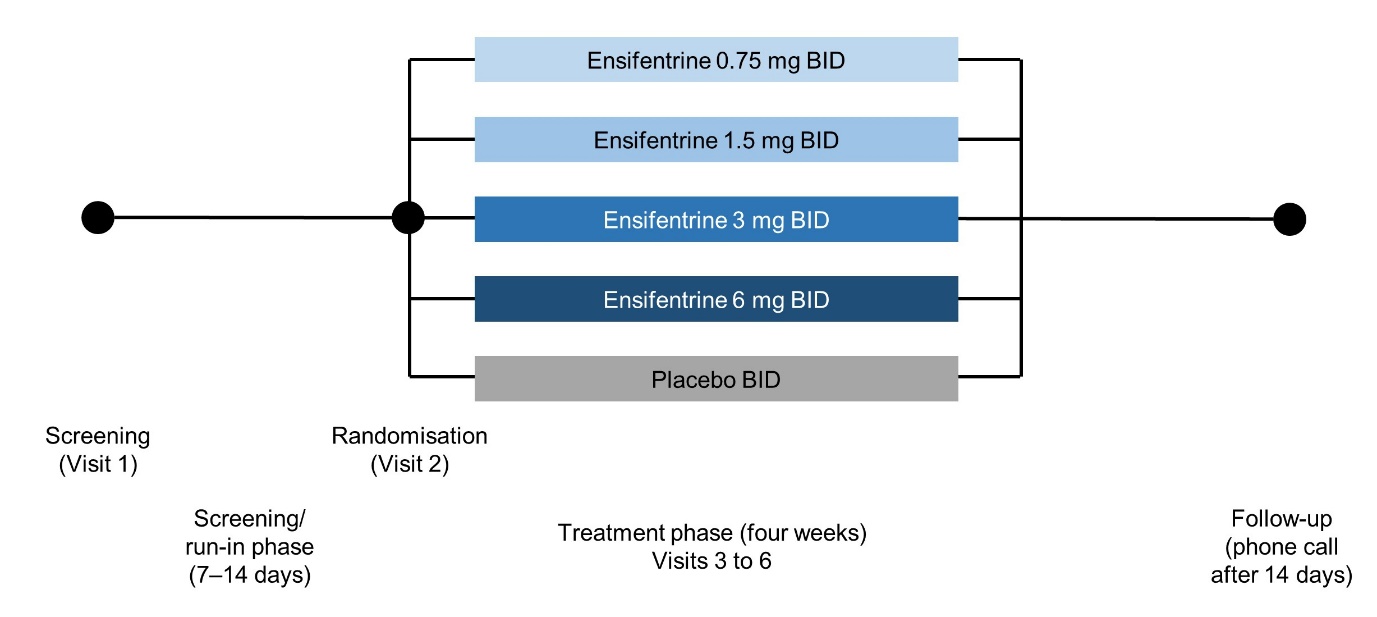


Abbreviation: BID, twice daily.

The protocol was amended twice. The first amendment added contraceptive requirements for non-sterile male participants and female participants of childbearing age, permitted the recruitment of current or former smokers, and added a number of vital signs and 12-lead electrocardiogram assessments. The second amendment clarified the wording of a number of exclusion criteria and permitted medications, corrected a number of inconsistencies, and clarified text on the use of rescue medication.

## Patients

### Inclusion criteria

Patients were eligible for inclusion into the study if they met each of the following criteria:

1. Signed an informed consent document indicating that they understood the purpose of and procedures required for the study and were willing to participate.
2. Male or female aged between 40 and 75 years inclusive, at the time of informed consent.
3. If male: was surgically sterile or had agreed to adhere to the following from the first dose up to the Telephone Follow-up, two weeks after the last dose of study medication:
   - Not donate sperm.
   - *Either*: was sexually abstinent in accordance with the patient’s usual and preferred lifestyle (but agreed to abide by the contraception requirements below should their circumstances change).
   - *Or:* used a condom with all sexual partners. If the partner was a female of childbearing potential, the condom was used with spermicide and a second reliable form of contraception (e.g., diaphragm/cap with spermicide, established hormonal contraception, intra-uterine device).

If female: was of non-childbearing potential, or agreed to use a highly effective form of contraception. All female patients of childbearing potential must have used this contraceptive from first dose until the Telephone Follow-up (2 weeks after final dose), and had a negative pregnancy test at Visit 1 and Visit 2 prior to randomisation.

1. Had a 12-lead electrocardiogram recording at Screening (Visit 1) which demonstrated the following (and no changes at Visit 2 deemed clinically significant by the Investigator):
   - Heart rate between 50 and 90 beats per minute (bpm).
   - QTc (Fridericia) interval ≤450 msec for males and ≤470 msec for females.
   - QRS interval ≤120 msec.
   - PR interval ≤200 msec.
   - No clinically significant abnormality including morphology (e.g., left bundle branch block, atrioventricular nodal dysfunction, or ST segment abnormalities consistent with ischemia).
2. Complied with all study restrictions and procedures, including ability to use the study nebuliser correctly.
3. Body mass index was between 18 and 35 kg/m^2^ (inclusive) with a minimum body weight of 45 kg.
4. Chronic obstructive pulmonary disease diagnosis: Patients with a COPD diagnosis as defined by the American Thoracic Society (ATS)/European Respiratory Society (ERS) guidelines and symptoms compatible with COPD for at least one year prior to Screening (Visit 1).
5. Was able to perform acceptable and reproducible spirometry. Post-bronchodilator (salbutamol 4 puffs) spirometry at Screening (Visit 1) must have demonstrated a:
   - Post-bronchodilator FEV_1_/forced vital capacity (FVC) ratio of ≤0.70.
   - Post-bronchodilator FEV_1_ ≥40% and ≤80% of predicted normal.
6. Clinically stable COPD in the four weeks prior to Screening (Visit 1) and Randomisation (Visit 2).
7. A chest X-ray (posterior-anterior) at Screening, or in the 12 months prior to screening showed no abnormalities that were clinically significant or related to COPD. A historical magnetic resonance imaging (MRI) or computed tomography (CT) scan in the 12 months prior to Screening (Visit 1) with equivalent results was accepted instead of a chest X-ray.
8. Met the concomitant medication restrictions for the duration of the study.
9. Current or former smoker with a smoking history of ≥10 pack-years.
10. For patients taking long-acting bronchodilators, capable of withdrawing from these medications until the end of the treatment period, and short acting bronchodilators for 8 h prior to administration of study medication.

### Exclusion criteria

Patients were excluded from the study if they met any of the following criteria:

1. A history of life-threatening COPD including admission to the Intensive Care Unit and the need for intubation.
2. COPD exacerbation that required oral steroids in the three months prior to Screening (Visit 1), or prior to Randomisation (Visit 2).
3. A history of 1 or more hospitalisations for COPD in the six months prior to Screening (Visit 1).
4. Lower respiratory tract infection treated with antibiotics within three months of Screening (Visit 1), or prior to Randomisation (Visit 2).
5. Evidence of cor pulmonale or clinically significant pulmonary hypertension.
6. Other respiratory disorders: patients with a current diagnosis of asthma, active tuberculosis, lung cancer, bronchiectasis, sarcoidosis, lung fibrosis, interstitial lung diseases, sleep apnoea, known alpha-1 antitrypsin deficiency or other active pulmonary diseases.
7. Previous lung resection or lung reduction surgery.
8. Oral therapies for COPD (e.g., oral steroids, theophylline, and roflumilast) in the three months prior to Screening (Visit 1) and throughout the study.
9. Pulmonary rehabilitation, unless such treatment had been stable from four weeks prior to Screening (Visit 1) and remained stable during the trial.
10. A history of, or reason to believe a patient had, drug or alcohol abuse within the past three years.
11. Received an experimental drug within 30 days or five half-lives of Visit 2, whichever was longer.
12. Prior exposure to ensifentrine.
13. Women who were pregnant or breast-feeding.
14. Patients with a history of current uncontrolled disease including, but not limited to, endocrine, thyroid disease neurological, hepatic, gastrointestinal, renal, haematological, urological, immunological, or ophthalmic diseases that the Investigator judged to be clinically significant.
15. Documented cardiovascular disease: arrhythmias, unstable angina, recent or suspected myocardial infarction within six months prior to Screening, congestive heart failure, a history of unstable or uncontrolled hypertension, or had been diagnosed with hypertension in last three months.
16. Use of oral beta-blockers.
17. Major surgery (requiring general anaesthesia) in the six weeks prior to Screening (Visit 1), lack of full recovery from surgery at Screening (Visit 1), or planned surgery through the end of the study.
18. History of malignancy of any organ system within five years, with the exception of localised skin cancers (basal or squamous cell).
19. Clinically significant abnormal values for safety laboratory tests (haematology, biochemistry or urinalysis) at Screening (Visit 1), as determined by the Investigator.
20. A disclosed history, or one known to the Investigator, of significant non-compliance in previous investigational studies or with prescribed medications.
21. Requirement for oxygen therapy, even on an occasional basis.
22. Any other reason that the Investigator considered would make the patient unsuitable to participate.
23. Known hypersensitivity to ensifentrine or its excipients/components.
24. Abnormal clinically significant 12 lead Holter findings, including but not limited to:
    - Premature ventricular contractions (PVCs) >1000 in 24-h period.
    - Sustained ventricular tachycardia >6 beats.
    - Atrial fibrillation with rapid ventricular response (>100 bpm).
    - Atrial flutter.
    - Sinus pause >2 seconds.

# Results

Table S1. Average FEV_1_ over 0–3 h (full analysis set).

| **Ensifentrine dose** | **Treatment–placebo difference, mL** | | | | |
| --- | --- | --- | --- | --- | --- |
|  | **Day 1** | **Week 1** | **Week 2** | **Week 3** | **Week 4** |
| 6 mg (N=80) | 150  (112, 188); <0.001 | 178 (120, 236); <0.001 | 160 (97, 223); <0.001 | 158 (94, 222); <0.001 | 133 (67, 198);  <0.001 |
| 3 mg (N=82) | 174 (136, 211); <0.001 | 205 (147, 263); <0.001 | 203 (141, 266); <0.001 | 209 (145, 273); <0.001 | 191 (126, 256); <0.001 |
| 1.5 mg (N=81) | 144 (106, 182); <0.001 | 184 (126, 242); <0.001 | 159 (96, 222); <0.001 | 183 (119, 247);  <0.001 | 150  (86, 215); <0.001 |
| 0.75 mg (N=81) | 126 (88, 163); <0.001 | 176 (118, 235); <0.001 | 148 (85, 211); <0.001 | 149 (85, 213);  <0.001 | 136  (70, 202); <0.001 |

Data are least squares mean treatment–placebo differences (95% confidence interval); p value. Least squares mean changes from baseline in the placebo group (N=79) were 17, –39, –28, –41 and –27 mL on Day 1 and at Weeks 1, 2, 3 and 4, respectively. Abbreviation: FEV_1_, forced expiratory volume in 1 second.

Table S2. Percentage of patients with ≥4 unit improvement in SGRQ-C total score after four weeks (full analysis set).

| **Treatment** | **Patients, %** | **Odds ratio vs placebo (95% confidence interval); p value** |
| --- | --- | --- |
| Ensifentrine 6 mg (N=80) | 51 | 1.75  (0.86, 3.55); 0.123 |
| Ensifentrine 3 mg (N=82) | 42 | 1.11  (0.53, 2.31); 0.791 |
| Ensifentrine 1.5 mg (N=81) | 32 | 1.76  (0.87, 3.56); 0.119 |
| Ensifentrine 0.75 mg (N=81) | 42 | 2.62  (1.29, 5.29); 0.008 |
| Placebo (N=79) | 26 |  |

Abbreviation: SGRQ-C, St George’s Respiratory Questionnaire – Chronic Obstructive Pulmonary Disease Specific.

Table S3. Medical Research Council dyspnoea score and patient global assessment of change after four weeks (full analysis set).

| **Ensifentrine dose** | **Treatment–placebo differences** | |
| --- | --- | --- |
|  | **Medical Research Council dyspnoea score** | **Patient global assessment of change** |
| 6 mg (N=80) | –0.08 (–0.31 to 0.14); 0.476 | 0.38 (0.14 to 0.63); 0.002 |
| 3 mg (N=82) | –0.10 (–0.32 to 0.13); 0.389 | 0.33 (0.09 to 0.58); 0.008 |
| 1.5 mg (N=81) | –0.13 (–0.35 to 0.10); 0.262 | 0.33 (0.09 to 0.58); 0.008 |
| 0.75 mg (N=81) | –0.09 (–0.31 to 0.14); 0.450 | 0.45 (0.20 to 0.70); 0.001 |

Data are least squares mean treatment–placebo differences (95% confidence interval); p value. Least squares mean changes from baseline in the placebo group (N=79) were –0.10 and 3.0 for Medical Research Council dyspnoea score and patient global assessment of change, respectively.

Table S4. Rescue medication use (full analysis set).

| **Ensifentrine dose** | **Treatment-placebo difference, puffs per day** | | | |
| --- | --- | --- | --- | --- |
|  | **Week 1** | **Week 2** | **Week 3** | **Week 4** |
| 6 mg (N=80) | –0.199 (–0.518 to 0.120); 0.221 | –0.601 (–1.006 to  –0.196); 0.004 | –0.591 (–1.007 to  –0.175); 0.006 | –0.567 (–0.988 to  –0.146); 0.008 |
| 3 mg (N=82) | –0.333 (–0.648 to  –0.019); 0.038 | –0.531 (–0.933 to  –0.129); 0.010 | –0.470 (–0.883 to  –0.057); 0.026 | –0.489 (–0.907 to  –0.071); 0.022 |
| 1.5 mg (N=81) | –0.115 (–0.430 to 0.200); 0.473 | –0.554 (–0.955 to  –0.153); 0.007 | –0.603 (–1.015 to  –0.191); 0.004 | –0.683 (–1.100 to  –0.266); 0.001 |
| 0.75 mg (N=81) | –0.069 (–0.387 to 0.250); 0.672 | –0.247 (–0.652 to 0.158); 0.231 | –0.330 (–0.746 to 0.087); 0.120 | –0.183 (–0.606 to 0.239); 0.395 |

Data are least squares mean treatment–placebo differences (95% confidence interval); p value. Least squares mean changes from baseline in the placebo group (N=79) were –0.491, –0.128, –0.085 and –0.127 for Weeks 1, 2, 3 and 4, respectively.
